# Association Between Intraindividual Variability in Cognitive Performance and White Matter Organisation in Chronic Mild Traumatic Brain Injury

**DOI:** 10.1002/hbm.70394

**Published:** 2025-11-01

**Authors:** Jake Burnett, Annalee L. Cobden, Alex Burmester, Hamed Akhlaghi, Juan F. Domínguez D, Karen Caeyenberghs

**Affiliations:** ^1^ Cognitive Neuroscience Unit, School of Psychology Deakin University Melbourne Australia; ^2^ Department of Emergency Medicine St Vincent's Hospital Melbourne Australia; ^3^ School of Psychological Sciences and Turner Institute for Brain and Mental Health Monash University Melbourne Australia

**Keywords:** cognition, diffusion MRI, ecological momentary assessment, fixel‐based analysis, intraindividual variability, traumatic brain injury, white matter

## Abstract

Mild traumatic brain injury (mTBI) can result in persistent cognitive deficits (particularly in attention, processing speed, and working memory), even years after the injury. The majority of behavioural studies have focussed on averaged cognitive performance scores, such as average reaction time or accuracy scores after mTBI. However, less is understood about how mTBI affects intraindividual variability (IIV) in cognitive performance across repeated sessions or measurement occasions over time. In this study, we investigate IIV in cognitive performance in chronic mTBI patients (*n* = 11) relative to healthy controls (*n* = 22). Participants underwent a single behavioural testing session (incorporating the Rivermead Post‐Concussion Symptom Questionnaire and a computerised processing speed task) and a multi‐shell diffusion MRI scan. This was followed by a 30‐day ecological momentary assessment (EMA) protocol using a smartphone app which measured symptoms and cognitive performance on a daily basis. Our results revealed that mTBI patients exhibited higher IIV than controls in both single‐session trial‐by‐trial and daily EMA measures. Higher daily IIV in cognitive performance coincided with higher daily fluctuations in post‐concussive symptoms. Additionally, mTBI patients showed reduced white matter organization, as indexed by fixel‐wise fibre density and fibre density cross‐section, in the left superior longitudinal fasciculus‐II compared to controls. Finally, trial‐by‐trial IIV was positively associated with white matter alterations in the SLF‐II in mTBI. Our findings suggest that mTBI results in dynamic performance deficits that persist into the chronic phase of injury. In addition, the white matter organization of a major fronto‐parietal tract seems to play an important role in supporting the consistency of cognitive performance over time, highlighting its potential as a biomarker for understanding cognitive dynamics in healthy adults and clinical populations.

## Graphical abstract

This study combined smartphone‐based ecological momentary assessment (EMA) and fixel‐based analysis (FBA) of diffusion MRI data to examine intraindividual variability (IIV) in cognitive performance and its neural correlates in chronic mild TBI. Our findings revealed persistent cognitive instability linked to daily symptom fluctuations and structural changes in the left superior longitudinal fasciculus‐II in mTBI patients.

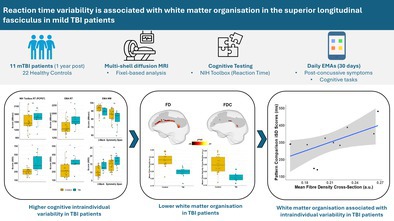

**Abbreviations**
ANTS: advanced normalisation tools
CC: corpus callosum
CFE: connectivity‐based fixel enhancement
CST: corticospinal tract
CT: computed tomography
DTI: diffusion tensor imaging
EMA: ecological momentary assessment
EPI: echo planar imaging
FA: fractional anisotropy
FBA: fixel‐based analysis
FC/logFC: (log)fibre cross‐section
FD: fibre density
FDC: fibre density cross‐section
FEW: family‐wise error
FOD: fibre orientation distribution
GCS: Glasgow Coma Scale
GLM: general linear models
IIV: intraindividual variability
iMean: intraindividual mean
iSD: intraindividual standard deviation
LOC: loss of consciousness
MD: mean diffusivity
MELSM: mixed effects location scale models
MRI: magnetic resonance imaging
MS3T‐CSD: multi‐shell 3‐tissue constrained spherical deconvolution
mTBI: mild traumatic brain injury
NART: National Adult Reading Test
NIH: National Institute of Health
PCPST: Pattern Comparison Processing Speed Test
RPQ: Rivermead Post‐Concussion Symptom Questionnaire
SIFT: spherical deconvolution‐informed filtering of tractograms
SLF: superior longitudinal fasciculus
WM: white matter

## 1 Introduction

Mild traumatic brain injury (mTBI) can often result in cognitive deficits, including difficulties with attention, memory, processing speed, and executive function, that can persist for months or years post‐injury (McInnes et al. 2017). In research, these deficits are typically quantified by comparing mTBI patients to a control group on the basis of average reaction time (RT) and/or accuracy scores using single administrations of one or more cognitive tests. For example, Mathias et al. (2004) showed that mTBI patients demonstrated slower mean RTs than controls on four visual RT tasks in the first month post‐injury, with their performance being more affected by increased task difficulty. However, this focus on differences in mean scores rests on the assumption that cognitive performance is a relatively stable and consistent attribute of an individual and overlooks the possibility that a person's performance might fluctuate significantly over time (Vaughan and Birney 2023). An alternative method for assessing cognitive deficits in mTBI involves the examination of *intraindividual variability* (IIV), or the inconsistency in an individual's performance over time or across different tasks (Hultsch et al. 2007).

A number of reports have suggested that mTBI is not only associated with slower or less accurate responses but is also characterized by heightened IIV in cognitive performance, particularly in the acute stages post‐injury. Early work by MacFlynn et al. (1984) provided the first empirical evidence of increased IIV in mTBI patients, demonstrating significantly greater variability in RTs across trials of a four‐choice RT task administered within 24 h of injury. Subsequent studies have reinforced these findings, showing acute increases in IIV in the first days following an mTBI (Cole et al. 2018; Green et al. 2019; Makdissi et al. 2001). For instance, Makdissi et al. (2001) observed that Australian Rules Football players with concussions exhibited both prolonged RTs and increased RT variability on a computerized simple RT task within 72 h post‐injury compared to non‐concussed controls. While these acute differences in IIV appear to diminish over time—often resolving by approximately 6 weeks post‐concussion (Beaupré et al. 2012; MacFlynn et al. 1984)—the long‐term trajectory of IIV deficits in mTBI remains poorly understood.

There is some limited evidence to suggest that increased IIV may persist into the chronic phase of injury in individuals with a more protracted recovery. A series of studies by Pearce et al. (2019, 2020, 2021) investigated RT IIV in chronic mTBI patients who were on average 1 year post‐injury. These studies consistently found that chronic mTBI patients with persistent symptoms demonstrated significantly slower and more variable RTs compared to asymptomatic patients. Notably, this heightened variability was also associated with higher self‐reported levels of mental fatigue. There is also evidence to suggest that residual IIV deficits may be present even in individuals who are considered “fully recovered” (Bleiberg 1997; Collins and Long 1996). For example, Collins and Long (1996) observed increased trial‐by‐trial IIV on simple and choice RT tasks in individuals with a history of mTBI, despite showing normal performance on standard neuropsychological measures such as the Impairment Index of the Halstead‐Reitan Neuropsychological Test Battery.

Until now, the majority of research investigating IIV in mTBI has measured IIV across trials during a single testing session. However, cognitive performance can fluctuate over a range of timescales, ranging beyond rapid, trial‐by‐trial variability over milliseconds or seconds to more enduring shifts in performance that unfold across “good” and “bad” days (Cerino et al. 2021). Investigating these enduring fluctuations in cognitive performance requires intensive longitudinal data collection methods involving frequent observations of each participant over extended periods that go beyond trial‐by‐trial measures (Sliwinski et al. 2018). Recent advancements in digital technology offer innovative solutions through the use of ecological momentary assessment (EMA) designs. EMA involves administering brief, frequent assessments of behaviours and experiences in naturalistic settings as individuals go about their daily lives (Shiffman et al. 2008). Using smartphone‐based applications, participants can respond to short surveys, record symptoms, or complete simple tasks at predetermined intervals.

EMA has been widely applied in behavioural research to track fluctuations in mood, stress, and fatigue, as well as other self‐reported symptoms over time. In the context of mTBI, EMA studies have primarily focused on monitoring recovery of post‐concussive symptoms based on daily symptom reports in the first weeks post‐injury (Pacella et al. 2018; Suffoletto et al. 2013; Sufrinko et al. 2019; Wiebe et al. 2022). Moreover, Juengst et al. (2019) investigated day‐to‐day variability in self‐reported emotional and fatigue symptoms in patients with mild‐to‐severe TBI. Their findings revealed significant temporal IIV in self‐reported symptoms, with higher IIV associated with greater overall symptom burden. Beyond self‐reported symptoms, ultra‐brief mobile cognitive tests embedded within EMA protocols offer a means of capturing multiple “snapshots” of an individual's cognitive performance over days or weeks. However, no studies to date have integrated cognitive tasks into EMA to measure cognitive performance variability in TBI.

In addition, a growing number of studies in healthy and clinical populations have linked IIV with structural brain characteristics, with particular involvement of white matter (WM) tracts implicated in attentional control and executive functioning (MacDonald et al. 2009). Several diffusion tensor imaging (DTI) studies in healthy cohorts have shown that increased IIV is associated with disruptions in WM microstructure, as reflected by reductions in fractional anisotropy (FA) and increases in mean diffusivity (MD) (Fjell et al. 2011; McCormick et al. 2023; Mella et al. 2013). For example, Fjell et al. (2011) showed that higher RT‐IIV was associated with reduced FA (covering > 25% of voxels) and increased MD (covering almost 50% of voxels) in a large lifespan cohort of healthy adults (aged 20 to 83 years). According to our knowledge, only one study has investigated the association between WM alterations and IIV in mTBI patients (Sorg et al. 2021). In this study, higher variability across different executive function tests was significantly associated with lower FA in the fornix, corpus callosum, and bilaterally in the cingulum, corticospinal tract, inferior fronto‐occipital fasciculus, and superior longitudinal fasciculus in military veterans with mTBI. However, this study employed the traditional DTI model, which cannot deal with crossing fiber populations and does not provide biologically specific metrics.

This study investigates IIV in cognitive performance in adults with chronic mTBI by combining daily smartphone‐based EMA (incorporating both cognitive tasks and self‐reported symptom measures), single timepoint lab‐based RT measures, and advanced diffusion MRI techniques. First, we compare IIV between mTBI patients and controls at two timescales, including trial‐by‐trial variability on a RT task administered in clinic and day‐to‐day variability captured over 30 days of EMA (measuring RT and working memory). We then evaluate whether cognitive IIV relates to self‐reported symptoms in the mTBI group. We also compare WM organisation between mTBI patients and controls using the novel fixel based analysis (FBA) framework and test whether the observed WM alterations are associated with IIV in mTBI patients. We hypothesise that (1) the mTBI group would show greater IIV at both timescales, (2) greater IIV would be associated with higher and more variable post‐concussive symptoms in the mTBI group, (3) the mTBI group would show reduced WM organisation using FBA, and (4) reduced WM organisation would be associated with increased IIV in mTBI.

## 2 Methods

### 2.1 Participants

A total of 11 mTBI patients (age range = 26 to 61, *M*_age_ = 40.27 [SD = 11.93]; 5 females) were recruited via the emergency department at St Vincent's Hospital in Melbourne, Australia. Eligible participants were identified retrospectively by study staff via screening of medical records and contacted via mail and phone for recruitment. Medical records were screened for inclusion criteria as follows: (1) age range between 18 and 65 years; (2) diagnosed with a mTBI (or a ‘concussion’) at the time of injury based on the American Congress of Rehabilitation Medicine (1993) definition, which includes: (i) a Glasgow Coma Scale (GCS) score between 13 and 15 at time of injury; (ii) loss of consciousness (LOC) under 30 min; (iii) post traumatic amnesia less than 24 h; (iv) any alteration in mental state at the time of the accident; (v) presence of focal neurological deficits; or (vi) where clinical information was not available, a clinician diagnosis of mTBI in the clinical notes; (3) time since injury of ≥ 3 months; (4) access to a smartphone with internet. The patients had sustained their mTBI approximately 1 year ago (*M* = 12.00 months, SD = 2.78, range 5–15 months). An overview of the demographic and clinical characteristics of the mTBI patients can be found in Table 1.

**TABLE 1:** Clinical characteristics of the mTBI patients.

ID	Age/sex	Months post‐injury	Mechanism	GCS	LOC	No. of previous mTBIs	CT results at time of injury	RPQ‐16 Total Score
TBI01	46/F	10.90	Fall	14	Yes	N/A	Normal	5
TBI02	31/M	11.10	Object vs. Head	15	No	N/A	Normal	28
TBI03	41/F	13.97	Fall	14	N/A	N/A	Normal	18
TBI04	28/M	12.13	Fall	N/A	Yes	N/A	Normal	43
TBI05	47/M	5.10	Fall	14	N/A	N/A	Normal	18
TBI06	61/M	11.87	Object vs. Head	15	Yes	1	Normal	16
TBI07	30/F	11.47	Fall	13	Yes	N/A	Normal	6
TBI08	33/F	14.03	Fall	14	Yes	2	Normal	27
TBI09	26/M	15.70	Sport	14	Yes	N/A	Normal	29
TBI10	42/F	11.37	Object vs. Head	15	Yes	1	Normal	39
TBI11	58/M	14.37	Assault	N/A	No	1	Normal	11

Abbreviations: CT = computed tomography, GCS = Glascow Coma Scale Score, LOC = loss of Consciousness, N/A = data not available or unknown.

A comparison group of 22 healthy controls (age range = 21 to 57 years; *M*_age_ = 34.36 [SD = 10.90]; 11 females) was recruited via social media advertisement and word‐of‐mouth channels. The healthy controls had to meet the following inclusion criteria: aged between 18 and 65 years and access to a smartphone. Exclusion criteria for both groups included any self‐reported history of: (1) a neurological disorder (besides previous concussion/mTBI for the mTBI group); (2) substance abuse; (3) severe psychiatric disorders (e.g., bipolar, schizophrenia; mild levels of depression and anxiety were not excluded); and (4) contraindications for MRI (e.g., ferromagnetic implants, metallic foreign bodies, claustrophobia, pregnancy). Two individuals in the healthy control group were excluded from MRI due to contraindications and were not included in the MRI analyses (*N*_MRI_ = 20).

Demographic characteristics of both groups are presented in Table 2. Participants predominantly held undergraduate degrees, and both groups exhibited above‐average premorbid intellectual functioning as measured by the National Adult Reading Test (NART; Nelson 1982). Furthermore, the sample was predominantly right‐handed, with the majority of participants self‐reporting right‐hand dominance.

**TABLE 2:** Demographic and clinical characteristics of the mTBI and control groups.

Variable	mTBI (*n* = 11)	Controls (*n* = 22)	*p* ^a^
Age (years)	40.27 (11.93)	34.36 (10.90)	0.18
Sex	6M/5F	11M/11F	1.00
Education			0.19
High School	2 (18.18)	4 (18.18)	
Professional Diploma	2 (18.18)	0 (0.00)	
Bachelor's Degree	4 (36.36)	14 (63.63)	
Postgraduate Degree	3 (27.27)	4 (18.18)	
NART IQ estimate, *M* (SD)	114.25 (5.30)	117.96 (4.48)	0.06
Handedness (right), *n* (%)	9 (81.81)	19 (86.36)	1.00

^a^ Group comparisons for demographic variables were performed using independent samples *t*‐tests for continuous variables (age, IQ) and Fisher's exact tests for categorical variables (gender, education, handedness).

### 2.2 Baseline Behavioural Assessment

#### 2.2.1 Post‐Concussion Symptom Questionnaire

We administered the Rivermead Post‐Concussion Symptom Questionnaire (RPQ), a validated tool designed to evaluate the presence and severity of post‐concussive symptoms in mTBI patients (King et al. 1995). The RPQ assesses 16 symptoms across several domains, including emotional (e.g., depression), cognitive (e.g., difficulty concentrating), somatic (e.g., fatigue), and physical symptoms (e.g., headaches, dizziness) (Table 3). The RPQ can be scored across two subscales that assess early symptom clusters often present in the acute phase (RPQ‐3) and late symptom clusters often present in the chronic phase (RPQ‐13). Participants rated the severity of each symptom they experienced over the past month using a 5‐point ordinal scale: 0 = “not experienced at all”, 1 = “experienced but not a problem” (or no more of a problem than pre‐injury), 2 = “mild problem”, 3 = “moderate problem”, 4 = “severe problem”. Total scores were calculated by summing the ratings across all items, resulting in a range in total score from 0 to 64, where higher scores indicate more severe symptomatology (see Table 1 for individual RPQ scores). Previous research has provided categorizations for interpretation of overall symptom severity based on RPQ‐16 total scores: 0–12 (minimal symptoms), 13–24 (mild symptoms), 25–32 (moderate symptoms), and > 33 (severe symptoms) (Potter et al. 2006).

**TABLE 3:** Symptoms of the Rivermead post‐concussion symptoms questionnaire‐16 (King et al. 1995).

RPQ‐3	RPQ‐13		
Physical	Cognitive	Emotional	Somatic
Headache	Difficulty concentrating	Irritable/angry	Sleep disturbance
Dizziness	Taking longer to think	Depressed/tearful	Fatigue
Nausea/vomiting	Forgetfulness	Frustrated/impatient	Double vision Blurred vision Light sensitivity Noise sensitivity

### 2.3 Neuropsychological Testing

Processing speed was assessed using the Pattern Comparison Processing Speed task (PCPST) of the computerised National Institute of Health (NIH) Toolbox Cognition Battery (Weintraub et al. 2013) on an iPad 6th Gen (9.7 in.). The NIH Toolbox‐Cognition battery is a multidimensional set of assessments used to measure cognitive function in people aged 3 to 85 and includes measures of executive function, attention, memory, language, and processing speed. In the PCPST, participants are presented with pairs of simple images on the screen and they are instructed to decide whether the pair of images are identical or different as quickly as possible. Participants have to respond by pressing a “yes” or “no” button to indicate whether the patterns are the same or different. Each pair of images remains visible for as long as it takes the participant to respond. Participants have to solve as many pairs as possible up to 130 trials. We extracted the raw trial‐by‐trial RTs (in milliseconds) for our analysis.

### 2.4 Ecological Momentary Assessment

EMA assessments were conducted using our in‐house developed smartphone application, *Mindtrax* (Burnett, Cobden, Burmester, et al. 2025; Cobden et al. 2025). Mindtrax allows for the repeated measurement of cognitive and psychological symptoms across time in various clinical populations. The app was downloaded onto the participant's personal device, and participants were provided with a demonstration of the app from the study personnel at the time of testing. The EMA component of the study commenced on the first evening following the in‐person testing session. Participants were instructed to complete the daily assessments (approximately 5 min per day) for 30 days in the evening at their own preferred time. A reminder notification was sent at 8:00 pm each night. Participants were reimbursed with a gift voucher at the end of the 30‐day study period. Each app session commenced with a questionnaire, followed by brief computerised cognitive assessments, which we describe in detail below.

#### 2.4.1 EMA Questionnaire

The EMA questionnaire included 13 symptoms adapted from the full RPQ according to the modified scoring system proposed by Eyres et al. (2005). The RPQ‐13 was selected due to its shorter length for EMA and coverage of late symptom clusters associated with the chronic phase of injury (Eyres et al. 2005). Participants were instructed to rate the severity of each symptom they experienced over the past 24 h using a 5‐point ordinal scale (i.e., scores ranging from 0 = not experienced at all, to 4 = severe problem). For each EMA session, we calculated domain‐specific and total scores for each participant. Total scores were calculated by summing the ratings across all items, ranging from 0 to 64, with higher total scores indicating more severe symptomatology. Domain‐specific scores were also calculated for each session for emotional symptoms (depressed, frustrated, irritability, restlessness), cognitive symptoms (forgetful, concentration problems, slowed thinking), and somatic symptoms (sleep disturbance, fatigue, blurred vision, double vision, light sensitivity, noise sensitivity).

#### 2.4.2 EMA Cognitive Tasks

**FIGURE 1:**
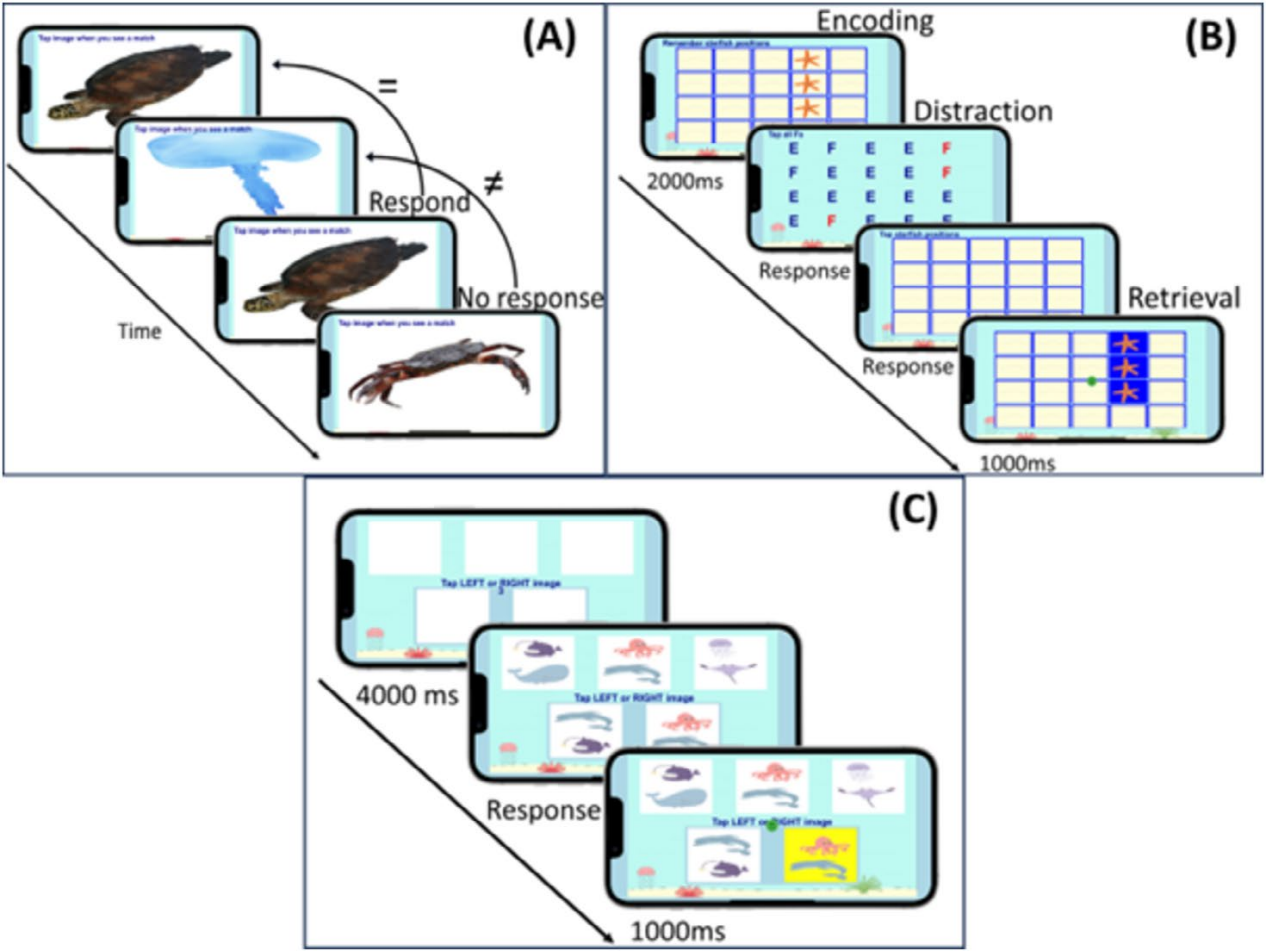
Overview of the MindTrax cognitive EMA tasks. (A) 2‐back task. Participants are presented with a series of images in sequential order and are required to tap the screen when the image presented is the same as the one two trials before. Each image was presented for 2000 ms. Participants completed 15 trials per session. (B) Symmetry span task. Participants were required to remember the spatial locations of three starfish randomly placed in a (4 × 5) grid for 2000 ms. After a distraction task (tapping on the F's amongst E's), the participants needed to indicate the original positions of the three starfish. Participants completed three of these trials each session, with 3 starfish to identify in each trial (9 in total). (C) Card matching task. A series of cards are displayed on the screen, with three cards on the top row and two cards on the bottom row. Participants were instructed to match one of the lower cards with one of the top cards as fast and as accurately as possible. The stimulus was presented until the participant responded. Each stimulus was preceded by a 4000 ms count down, presented as ‘get ready’, ‘3’, ‘2’, ‘1’, which were all displayed for 1000 ms each. Participants completed 10 trials per session.

EMA cognitive tasks included (1) a computerised 2‐back task (assessing visual executive working memory), (2) a Symmetry Span task (assessing visuospatial working memory), and (3) a Card Matching Task (assessing attention/processing speed) (see Burnett, Cobden, Burmester, et al. 2025; Cobden et al. 2025). Each task took approximately 1 to 2 min to complete. A detailed overview of the EMA tasks is presented in Figure 1. For the working memory tasks, total accuracy of responses was calculated for each session (as a percentage from 0 to 100%). For the Card Matching task, average RTs across trials were calculated for each session.

### 2.5 Compliance

Compliance with the EMA protocol was calculated as the total number of EMA sessions completed divided by the total number of prompts administered (i.e., 30), expressed as a percentage. Given the exploratory nature of our study, we applied a 50% compliance threshold for inclusion. While lower than typical EMA compliance thresholds, this decision was made to accommodate the flexible nature of the study design and to allow for the inclusion of participants who may have faced challenges completing the full protocol. Importantly, all participants met this threshold. Across all 33 participants, a total of 775 daily sessions were recorded by the EMA period over the 30‐day measurement period. The mTBI group completed a total of 251 daily sessions, with an average of 22 (SD = 4.40, range = 16–29) completed sessions across participants, yielding a mean compliance rate of 76.06% (SD = 14.66, range: 53%–97%). The control group completed a total of 524 sessions, with an average of 23 completed sessions across participants (SD = 3.19, range = 18–29) and an average compliance rate of 79.24% (SD = 10.63, range: 60%–97%). There were no significant differences between the groups in compliance rates, *t*(31) = 0.71, *p* = 0.48. In addition, there was no association between compliance rates and severity of mTBI symptoms measured by the EMA RPQ‐13 Total scores (*p* = 0.34). Technical issues with the app server resulted in missing data for a few trials on specific tasks for 7 participants in the control group; however, the amount of missing data was minimal (less than 1% of data missing overall) and had negligible impact on the overall dataset.

### 2.6 Calculation of Intraindividual Variability Metrics

To investigate individual differences in variability, we calculated the intraindividual mean (iMean) and standard deviation (iSD), which are commonly used in EMA studies to capture the dynamic nature of psychological and behavioural processes within individuals (Ram and Gerstorf 2009). Specifically, the iSD and iMean were calculated for each participant for each variable of the EMA tasks. The iMean represents the average score for a given variable across all time points for a single participant, providing a measure of their typical level of performance or symptomatology. The iSD represents the standard deviation of these scores, reflecting the overall magnitude of fluctuation or variability in the participant's responses over time (Ram and Gerstorf 2009). Prior to computing IIV metrics, the raw data were assessed for outliers using extremity scores to remove observations that fell outside of ±3.29 standard deviations of the respective group mean.

## 3 Magnetic Resonance Imaging

### 3.1 MRI Acquisition

MRI scans were acquired on a 3T Siemens PRISMA scanner with a 64‐channel head coil at the Florey Institute of Neuroscience and Mental Health (Heidelberg, Melbourne). Multi‐shell diffusion‐weighted imaging was acquired using a single‐refocused echo planar imaging (EPI) sequence with the following parameters: 84 axial slices; 1.8 mm isotropic voxels; TE/TR = 98 ms/3275 ms; flip angle = 90°; acquisition matrix = 128 × 128; multiband acceleration factor = 4; phase encoding anterior–posterior (AP); and SENSE1 multi‐coil reconstruction. The diffusion‐weighting scheme included *b* values = 0, 1600, 5000 with 8/25 / 64 volumes, respectively. Half of the volumes were acquired in the A>>P and half in the P>>A phase encoding directions. Additionally, we also acquired the corresponding phase images for complex data denoising (Cordero‐Grande et al. 2019). The overall duration of the DWI scan was approximately 6 min.

### 3.2 MRI Pre‐Processing

Before analysing the dMRI data, we conducted a thorough quality assessment of the raw dMRI images (e.g., “looping” through the DWI volumes, checking artefacts, color‐coded FA map, residuals and outlier profiles). All data were considered good quality for further analysis. The MRI data pre‐processing and analyses were performed using MRtrix3 (V3.0.4), in line with our previous work (Clemente et al. 2021; Liang et al. 2021; Verhelst et al. 2019) and MRtrix3 documentation (https://3Tissue.github.io). Pre‐processing steps included: complex denoising (using phase images; Cordero‐Grande et al. 2019), removal of Gibbs ringing artefacts (Kellner et al. 2016), and eddy current, motion, and susceptibility induced distortion correction with outlier replacement (Andersson et al. 2016; Andersson and Sotiropoulos 2016; Smith et al. 2004). Bias field correction was then performed based on the b0 data using the Advanced Normalization Tools (ANTS) N4 algorithm (Avants et al. 2009). The pre‐processed dMRI images were then upsampled from 2.5 to 1.25 mm isotropic voxels, followed by computation of upsampled brain masks.

### 3.3 Fibre Orientation Distribution Calculation

Following these preprocessing steps, individual fibre orientation distribution (FOD) maps were computed using multi‐shell 3‐tissue constrained spherical deconvolution (MSMT‐CSD; Jeurissen et al. 2014), using group‐averaged response functions for WM, grey matter (GM), and CSF (Dhollander et al. 2016, 2019). We then performed joint bias field correction and global intensity normalisation to ensure comparability of FOD amplitudes across subjects (Dhollander, Tabbara, et al. 2021; Raffelt, Tournier, et al. 2017). A study‐specific unbiased FOD template was generated using the FOD templates from all participants using linear and non‐linear registration of the FOD image (Raffelt et al. 2011; Raffelt, Tournier, Crozier, et al. 2012). Each individual FOD image was registered to the template space. FOD segmentation was then performed to compute fixels at the template and individual level (Raffelt et al. 2015; Raffelt, Dhollander, et al. 2017). Individual‐level fixels were reoriented to the corresponding fixels of the FOD template for group comparison of fixel‐wise metrics (Raffelt et al. 2015; Raffelt, Dhollander, et al. 2017).

### 3.4 Fixel Metrics Calculation

Fixel metrics were computed for each participant across all WM fixels. These included fibre density (FD; a microstructural measure of axonal density in a given fibre population), (log) fibre cross‐section (logFC; a macrostructural measure of the cross‐sectional area occupied by a given fibre bundle), and fibre density cross‐section (FDC; a combined measure of FD/FDC reflecting the tract's capacity for information transmission) (Dhollander, Clemente, et al. 2021; Raffelt, Dhollander, et al. 2017). This produced whole‐brain FD, logFC and FDC fixel maps for each participant. These three statistics were utilised for further analyses.

### 3.5 Tract of Interest Construction

**FIGURE 2:**
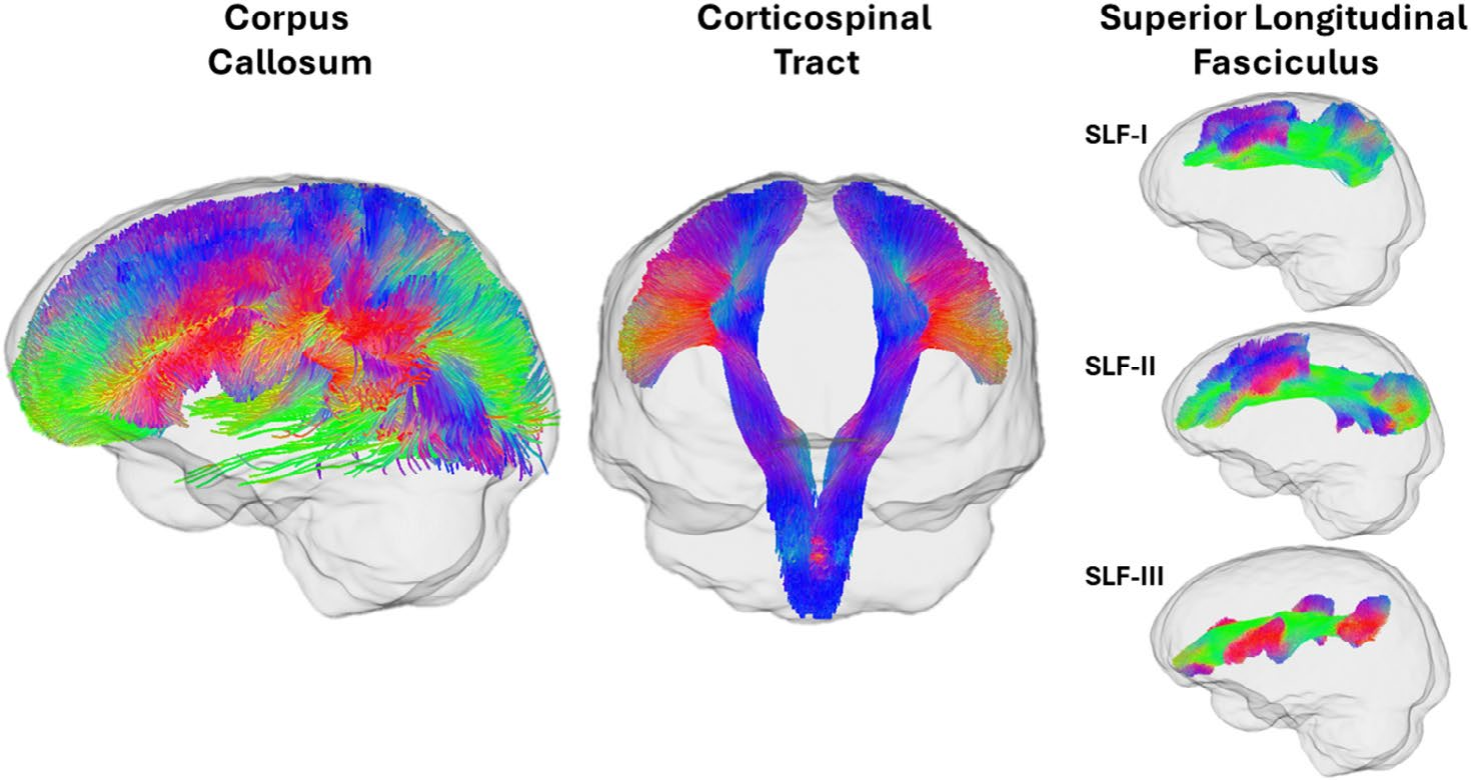
Glass brain depicting the corpus callosum, corticospinal tract and superior longitudinal fasciculus (I, II, and III). Tracts were delineated using TractSeg (Wasserthal et al. 2018, 2019). Tractograms from the left and right hemispheres were concatenated to generate a single, bilateral tractogram for each tract.

We used the TractSeg tool to delineate our tracts‐of‐interest. TractSeg can be used for semi‐automated probabilistic tractography, providing fast and accurate segmentation of complex WM bundles from diffusion MRI data (Wasserthal et al. 2018, 2019). TractSeg operates by first estimating a continuous streamline tractogram using probabilistic tractography algorithms. Subsequently, the tractogram is segmented into individual fibre bundles based on learned features from a convolutional neural network. We applied TractSeg to the study‐specific population template to segment those voxels corresponding to three tracts‐of‐interest: superior longitudinal fasciculus (three branches; SLF‐I, SLF‐II, SLF‐III), corpus callosum (CC), and corticospinal tract (CST) (Figure 2). These tracts were selected based on previous evidence reporting that these are among the most consistently reported sites of WM alterations following mTBI (see reviews Lindsey et al. 2023; Patil et al. 2025; Shenton et al. 2012) Moreover, these tracts collectively provide representative coverage of major commissural, projection, and association pathways vulnerable to injury. Finally, alterations in these tracts have been associated with cognitive performance variability in prior DTI studies in both healthy and clinical populations (e.g., Anstey et al. 2007; Burnett, Cobden, Burmester, et al. 2025; Halliday et al. 2019; Wolfers et al. 2015). Tractograms were concatenated across hemispheres to produce a bilateral tractogram for each tract. These tractograms were then applied to each individual participant's whole‐brain fixel maps to specifically crop the fixels belonging to the SLF, CC and CST. These individual (cropped) fixel maps were then submitted for statistical analyses.

## 4 Statistical Analyses

To examine differences between the groups, we used non‐parametric methods due to the non‐normal distribution of our variables. Specifically, we employed Mann–Whitney U tests to compare average performance (iMean scores) and variability (iSD scores) between the mTBI and control groups for each task. To quantify the effect size, rank biserial correlations (*r*_pb_) were calculated, which provide an estimate of the strength of the association between group membership and the rank order of the scores. Effect sizes were interpreted using conventional benchmarks (Cohen 1988), with values of 0.10, 0.30, and 0.50 representing small, medium, and large effects, respectively.

Spearman's correlation analysis was used to investigate the relationships between IIV, mean performance, and symptomatology within our mTBI group. Specifically, we performed the following two sets of correlation analyses: (1) the association between day‐to‐day cognitive IIV and day‐to‐day symptom severity (as measured via iMean and iSD scores on the EMA RPQ‐13), and (2) the relationship between single timepoint trial‐by‐trial variability (on the PCPST) and overall symptom severity (as measured by total scores on the RPQ‐16 in the clinic). All behavioural analyses were conducted in R (version 4.3.3).

To identify group differences in fixel metrics, we used the connectivity‐based fixel enhancement (CFE) method in MRtrix3 (Raffelt et al. 2015). CFE provides a permutation‐based (5000 permutations), family‐wise error (FWE) corrected *p* value for each individual fixel in the population template space. This analysis was performed for the a priori tracts‐of‐interest (CC, CST, and three branches of the SLF). The statistical significance threshold for group differences was set at *p*_FWE_ < 0.05. We applied a threshold to the FWE‐corrected *p* value maps to include only fixels with a corrected *p* value less than 0.05. Using this thresholded image, we conducted CFE‐based general linear models (GLMs) to examine the association between IIV variables (which showed significance in the group differences on the cognitive tasks) and fixel metrics of the tracts‐of‐interest (using the resulting significant fixels of the group analyses). Associations between fixel metrics and IIV were assessed using CFE (Raffelt et al. 2015), both before and after controlling for age, NART estimated IQ, and time since injury. Analyses were performed on the bilateral SLF tractogram using the default CFE smoothing parameters in MRtrix3 (smoothing = 10 mm full‐width at half maximum, *C* = 0.5, *E* = 2, *H* = 3). As per MRtrix3 recommendations, predictors and covariates (IIV, age, IQ, time since injury) were mean‐centred (demeaned) prior to model estimation. We conducted separate directional contrasts to test both positive and negative associations with IIV.

## 5 Results

### 5.1 Intraindividual Variability in Cognitive Performance

**FIGURE 3:**
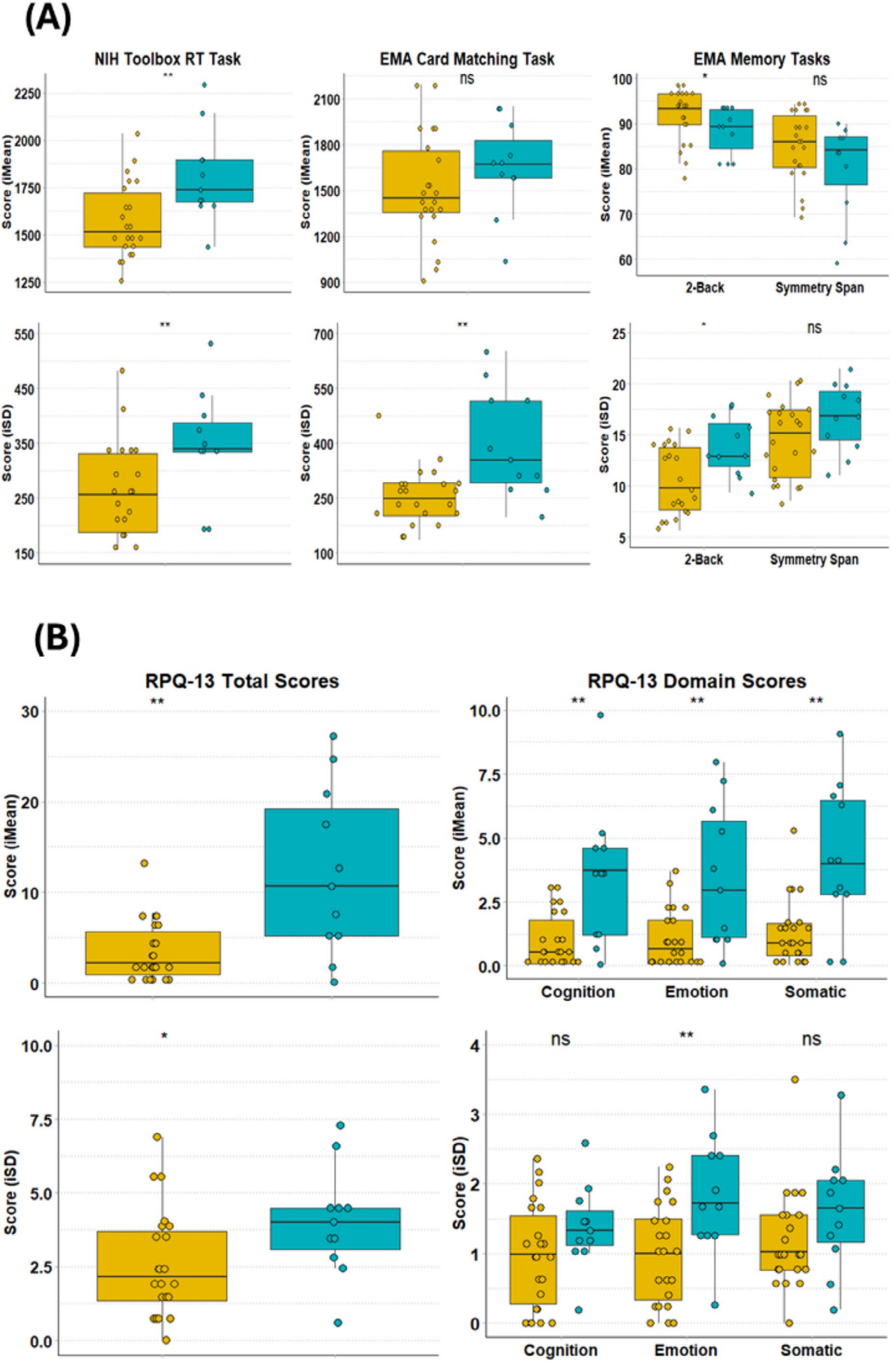
Boxplot of group differences in iMean and iSD scores between mTBI patients and controls on (A) the NIH Toolbox PCPST and EMA cognitive tasks; and (B) EMA RPQ‐13. Average scores (iMean scores) are displayed in the top row, and iSD scores are displayed in the bottom row. *p ≤ 0.05, **p ≤ 0.01, ns = not significant.

Performance on the EMA cognitive tasks revealed several significant group differences. With regard to the EMA processing speed task (Card Matching), the mTBI group showed significantly higher IIV in RTs compared to the controls, *W* = 46, *p* = 0.004, with a moderate effect size (*r*_pb_ = 0.49). For the visual executive working memory task (2‐back), the mTBI group showed significantly lower overall accuracy compared to controls, *U* = 173, *p* = 0.04; moderate effect size, *r*_pb_ = 0.34. Additionally, the mTBI group showed significantly higher IIV over time compared to the controls, *U* = 55, *p* = 0.01, with a moderate effect size (*r*_*pb*_ = 0.43). No significant differences in mean scores or IIV were observed between the groups on the EMA visuospatial working memory task (Symmetry Span). Examination of single‐session trial‐by‐trial performance on the NIH Toolbox PCPST showed that the mTBI group performed significantly more slowly compared to controls, *U* = 53, *p* = 0.009, with a moderate effect size (*r*_pb_ = 0.45). Additionally, the mTBI group showed significantly higher IIV in RTs across successive trials compared to controls, *U* = 51, *p* = 0.007, with a moderate effect size (*r*_pb_ = 0.46). Descriptive statistics of the IIV metrics are provided in Table 4. Boxplots illustrating the group differences in mean performance and IIV across the tasks are visually represented in Figure 3a.

**TABLE 4:** Descriptive statistics of intraindividual variability metrics across the groups.

Task	mTBI (*M*, SD)	HC (*M*, SD)
EMA card matching (iMean, ms)	1654.36 (298.61)	1514.54 (352.56)
EMA card matching (iSD, ms)	397.02 (147.21)	252.31 (78.15)
EMA 2‐back (iMean, %)	88.42 (5.25)	91.65 (5.81)
EMA 2‐back (iSD, %)	13.83 (2.89)	10.26 (3.52)
EMA symmetry span (iMean, %)	80.12 (10.46)	85.02 (7.57)
EMA symmetry span (iSD, %)	16.79 (3.31)	14.58 (3.67)
NIH pattern comparison (iMean, ms)	1807.59 (242.59)	1574.00 (200.47)
NIH pattern comparison (iSD, ms)	347.59 (96.66)	257.17 (89.46)

### 5.2 Associations Between IIV and Symptomatology

On the EMA symptom questionnaire (RPQ‐13), mTBI patients reported significantly higher intraindividual mean (iMean) daily symptom levels across all symptom domains compared to controls (Figure 3b). Specifically, mTBI patients reported elevated mean levels of emotional symptoms (*U* = 51, *p* = 0.007, *r*_*pb*_ = 0.46), cognitive symptoms (*U* = 41, *p* = 0.002, *r*_*pb*_ = 0.53), somatic symptoms (*U* = 49, *p* = 0.006, *r*_*pb*_ = 0.47), and total symptoms (*U* = 49, *p* = 0.006, *r*_*pb*_ = 0.47) compared to controls. Analysis of iSD scores revealed that mTBI patients demonstrated significantly greater variability in emotional symptoms (*U* = 53, *p* = 0.009, *r*_*pb*_ = 0.45) and RPQ‐13 total symptoms (*U* = 67, *p* = 0.04, *r*_*pb*_ = 0.35) compared to controls (Figure 3b). Variability in cognitive and somatic symptoms did not differ significantly between groups (*p* = 0.10 and 0.08, respectively).

**FIGURE 4:**
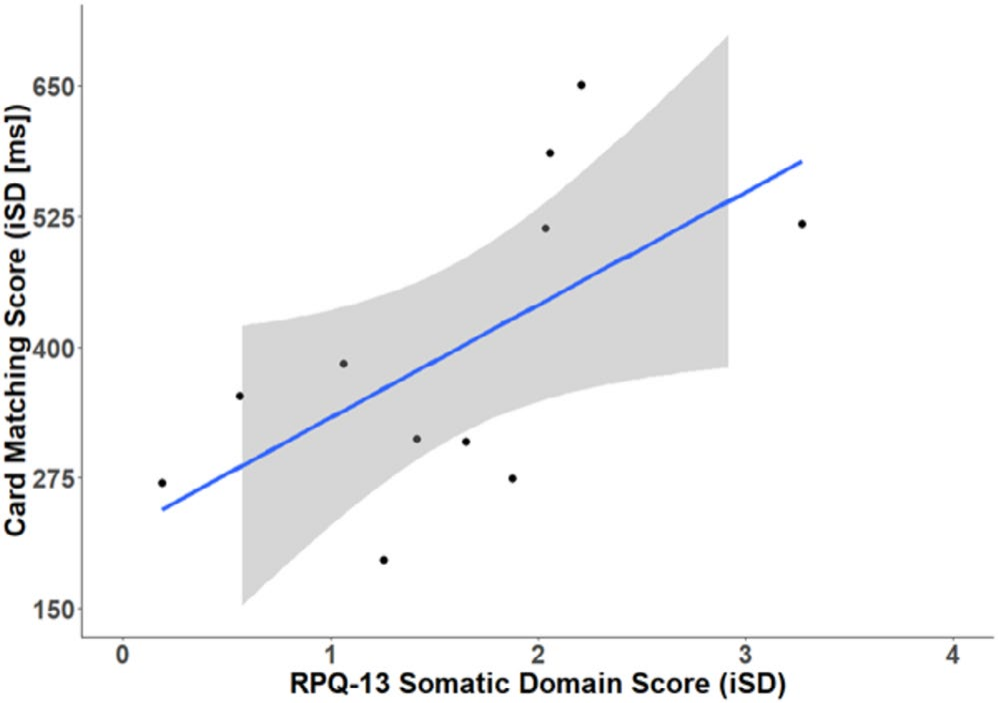
Scatterplot of the significant correlation between variability scores for the EMA‐adapted RPQ‐13 somatic symptom domain and variability scores on the EMA Card Matching RT task in the mTBI group.

Next, we examined the association between IIV in cognitive performance and symptomatology in the mTBI group. Spearman's correlation analyses revealed a significant positive relationship between IIV on the EMA Card Sorting RT task and somatic symptom scores on the EMA RPQ‐13 (*ρ* = 0.69, *p* = 0.01) (Figure 4). No other associations were observed between EMA symptom scores and EMA cognitive IIV for the mTBI group. A complete correlation matrix is provided in the Table S1. No associations were observed between trial‐by‐trial IIV on the PCPST and symptoms measured using the RPQ‐16 questionnaire at a single timepoint (*p* > 0.05).

### 5.3 Tract‐of‐Interest Analysis

**FIGURE 5:**
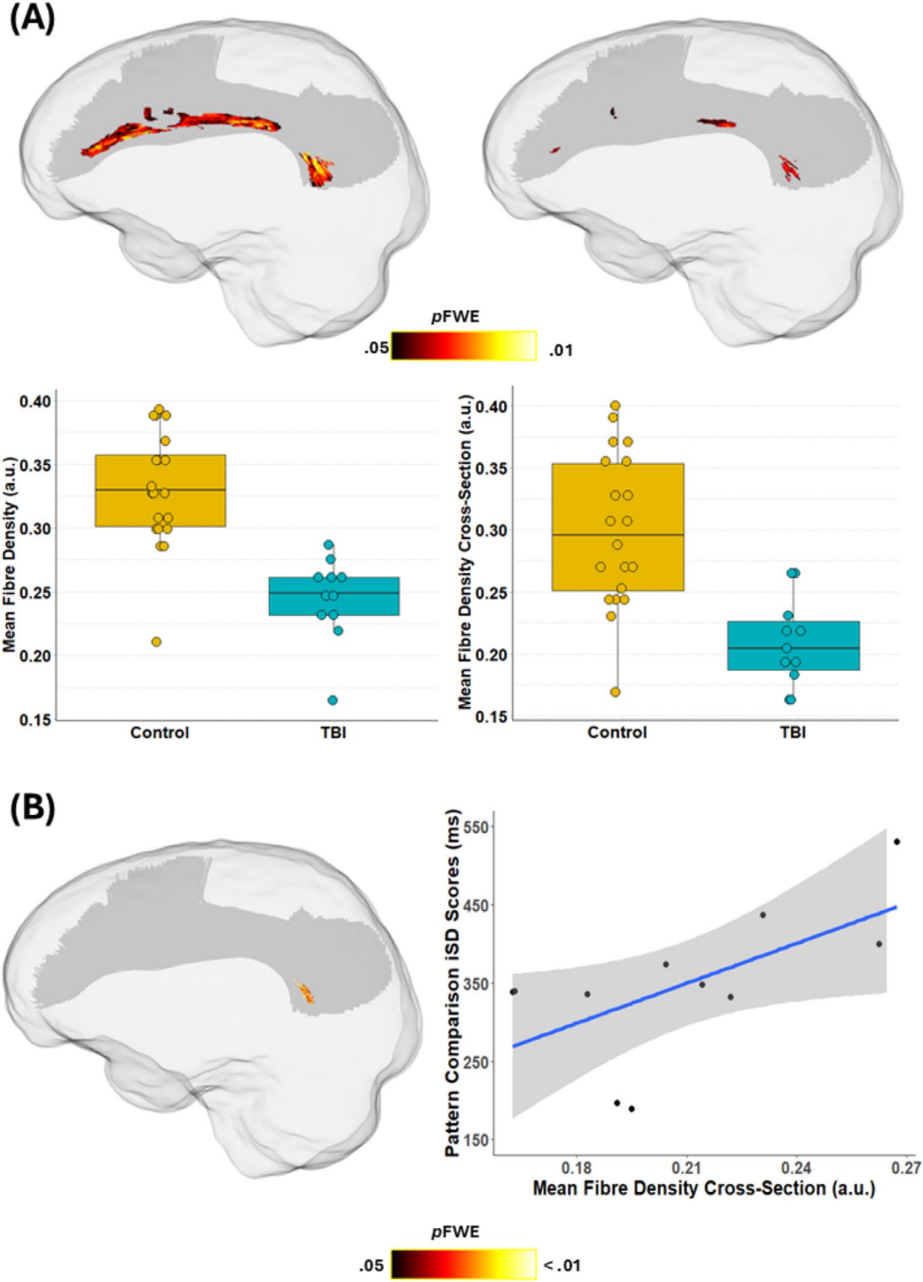
(A) Glass brain depicting groupwise comparisons of fibre density and cross‐section of the left SLF‐II between the mTBI (*n* = 11) and control (*n* = 20) groups, with FWE‐corrected *p* value map (top row) with boxplots showing group differences in mean fixel metrics of the significant clusters. (B) Glass brain depicting left SLF‐II fixels that showed a significant association with PCPST iSD scores in the mTBI group (left) with a scatterplot of the association (right) a.u. = arbitrary units.

Our tract‐of‐interest analysis revealed significant microstructural and macrostructural alterations among mTBI patients compared to healthy controls. Specifically, we observed statistically significant reductions in both FD (minimum *p*_*FWE*_ = 0.02) and FDC (min. *p*_*FWE*_ = 0.04) metrics in the left SLF‐II in mTBI patients (Figure 5a). A substantial decrease in microstructural FD was observed in the left SLF‐II, with a median FD reduction of 30% (range: 14.69%–84.02%) in mTBI patients compared to controls. When examining the combined microstructural and macrostructural characteristics using the FDC metric, the SLF‐II exhibited median FDC reductions of 38% (range: 24.14%–72.08%). Notably, the spatial distribution of these FDC reductions closely corresponded with clusters of reduced FD (Figure 5a). No other tracts showed statistically significant differences between mTBI patients and controls after family‐wise error correction.

Because the observed reductions were localized in the left hemisphere, we calculated a lateralisation index (LI) as follows: LI = (R−L)/(R + L). In this way, LI values range from −1 to +1, where negative values indicate left‐hemisphere dominance and positive values indicate right‐hemisphere dominance (Bianco et al. 2023; Vingerhoets et al. 2023). Values close to 0 indicate little or no lateralisation, and scores approaching ±1 indicate strong absolute hemispheric lateralisation. Across both groups, average laterality indices were close to zero for both FD (*M*_TBI_ = −0.03, SD_TBI_ = 0.01; *M*_control_ = −0.04, SD_Control_ = 0.01) and FDC (*M*_TBI_ = −0.02, SD_TBI_ = 0.03; *M*_control_ = −0.03, SD_Control_ = 0.02). Between‐group comparisons using Welch's t‐tests showed no significant group differences in laterality for FD (*t*(20.67) = −1.41, *p* = 0.17, *d* = 0.55) or FDC (*t*(15.94) = −1.53, *p* = 0.14, *d* = 0.65).

### 5.4 Associations Between Fixel Metrics and IIV

Next, we explored the bivariate associations between IIV and WM organisation, based on the significant differences we observed between the groups on the cognitive tasks (i.e., 2‐back, Card Matching and PCPST) and significant fixels of the left SLF‐II (*p*_FWE_ < 0.05). The GLM analysis showed that higher trial‐by‐trial variability on the PCPST was associated with higher FDC values of the SLF‐II for the mTBI group (min. *p*_FWE_ = 0.009), but not for controls. This cluster remained marginally significant after controlling for age, NART IQ, and time since injury in the covariate‐adjusted model (min. *p*_FWE_ = 0.04). To quantify the strength of this relationship, we extracted each participant's mean FDC values of the significant fixels in the SLF‐II and computed correlation coefficients with the PCPST iSD scores. This correlation analysis showed a moderate positive association between trial‐by‐trial IIV on the PCPST and FDC of the SLF‐II for the mTBI group (*r*(11) = 0.62, Figure 5b). No significant associations were observed between IIV on the 2‐back or Card Matching task and fixel metrics of the SLF‐II in either of the groups.

## 6 Discussion

The current study explored the nature of IIV in cognitive performance among individuals with chronic mTBI compared to healthy controls, along with its relationship to symptom fluctuations and WM integrity. To our knowledge, the present study is the first to (1) examine cognitive IIV in individuals with mTBI over an extended period in naturalistic settings using smartphone‐based EMA, and (2) examine the association between these performance fluctuations and symptomatology and WM organisation in mTBI patients.

### 6.1 Increased Intraindividual Variability in Chronic mTBI Patients

This study extends the current understanding of IIV by demonstrating persistent cognitive instability into the chronic phase of mTBI. A number of prior studies have observed increases in IIV after mTBI, with many studies documenting heightened performance variability in the first few days or weeks post‐injury (Cole et al. 2018; MacFlynn et al. 1984; Makdissi et al. 2001). In most cases, this instability gradually resolves alongside the amelioration of symptoms over the first 3 months of injury (Beaupré et al. 2012). Our patient group, who were on average 1 year post‐injury, exhibited significant IIV in performance on measures of visual RT and working memory compared to healthy controls. Our findings suggest that cognitive instability is not merely a transient phenomenon of the acute recovery period but may serve as a marker of long‐term, chronic dysfunction in mTBI.

The existing literature on variability in cognitive performance in mTBI patients has primarily measured IIV as trial‐level fluctuations in speeded responses on tasks administered in controlled laboratory settings (e.g., Makdissi et al. 2001). By utilizing a smartphone‐based EMA design, we were able to expand this approach to capture performance fluctuations on a daily basis over a one‐month period in naturalistic settings. We demonstrate that day‐to‐day variability in RTs, a longer timescale than traditional trial‐level approaches, is also sensitive to mTBI. The findings of the present study are broadly consistent with prior laboratory‐based research demonstrating significant trial‐level RT variability in TBI patients compared to controls (e.g., Makdissi et al. 2001; Stuss et al. 1994, 2003). Our findings suggest that mTBI is not only associated with increased RT variability at the trial level but is also characterized by broader and more pervasive patterns of cognitive instability, as evidenced by the substantial levels of RT variability observed over 30 repeated daily assessments.

Most studies examining IIV in mTBI have focused on simple and choice RT tasks that primarily assess basic visual recognition and signal detection (e.g., Cole et al. 2018; Green et al. 2019; Makdissi et al. 2001; Sosnoff et al. 2007). This is due, in part, to the inherent challenges of quantifying fluctuations in higher‐order cognitive processes (e.g., executive functioning or working memory) using conventional methods. For example, working memory tasks often quantify performance based on composite measures of accuracy (e.g., percentage correct, errors, misses/false alarms), usually derived from a smaller number of trials compared to RT tasks (Hultsch et al. 2007). As a result, intensive data collection involving repeated administration of the same task over an extended period is necessary to fully capture working memory variability (Sliwinski et al. 2018). Our EMA‐based cognitive tasks provided a unique opportunity to capture IIV in visual working memory across multiple sessions over a 30‐day period. Although mTBI patients were less consistent and performed slightly worse than controls, both groups achieved an average accuracy above 85%, suggesting that our study sample was generally high‐functioning in this domain. Nonetheless, the differences we detected illustrate the potential of EMA to reveal even subtle disruptions to cognitive stability in mTBI patients.

The use of EMA‐based post‐concussive symptom reports also provided an opportunity to examine how daily self‐reported symptoms were related to IIV on the EMA cognitive tasks. Our findings build on previous reports that patients with persistent post‐concussion symptoms exhibit higher IIV compared to asymptomatic patients (Pearce et al. 2019, 2020, 2021). We found that daily variability on the EMA Card Matching task was positively associated with fluctuations in daily RPQ‐13 somatic symptoms. In other words, patients with greater symptom fluctuations also showed more variability in cognitive performance. With our study as a foundation, a natural progression for future research is to examine the temporal structure of these relationships; that is, whether shifts in symptoms precede changes in cognitive performance, follow them, or occur concurrently (Ebner‐Priemer et al. 2009). Addressing this question will require sophisticated methods capable of capturing the sequencing of these fluctuations, such as time‐lagged or autoregressive models (Ram and Gerstorf 2009). Such analyses would require a denser sampling scheme, ideally with multiple assessments throughout the day (Ebner‐Priemer et al. 2009), which was not feasible in our study's daily diary design.

Our work helps address an open question as to whether repeated smartphone‐based cognitive tests embedded within an EMA protocol can be used to track fluctuations in cognitive performance in mTBI compared to controls (for a detailed review, see Burnett, Cobden, Firman‐Sadler, et al. 2025). The pronounced variability we observed across different domains and temporal scales suggests a broad and pervasive pattern of cognitive and symptom instability in chronic mTBI patients. This problem has direct implications for treatment evaluation. For example, when evaluating treatment response or recovery trajectories, patients with high IIV may require larger changes in mean performance to demonstrate reliable improvement that exceeds the bounds of their typical variability, whereas smaller mean changes in low‐IIV individuals may be more clinically meaningful (Bigler et al. 2024; Juengst et al. 2019). Moreover, an apparent “recovery” based on mean scores from a single timepoint may simply reflect a period of relatively high functioning within a patient's fluctuating performance range, leading to premature conclusions about symptom resolution (Bleiberg 1997; Collins and Long 1996). This suggests that cognitive deficits in mTBI can be better captured using a range of test scores acquired over multiple sessions across an extended period (e.g., over several weeks with EMA), rather than being represented by a single timepoint measurement (Burnett, Cobden, Burmester, et al. 2025; Burnett, Cobden, Firman‐Sadler, et al. 2025).

### 6.2 Fibre‐Specific Reductions in White Matter Organisation in Chronic mTBI

Previous research has largely relied on DTI to examine WM changes following mTBI (Lindsey et al. 2023; Shenton et al. 2012). While these studies have reported persistent alterations (such as decreased FA or increased MD) up to several years post‐injury (Lindsey et al. 2023), DTI‐derived metrics like FA and MD are limited in their specificity, as they are influenced by a range of biological and geometric factors (e.g., crossing fibres) (Farquharson et al. 2013). In contrast, the current study employed FBA, which models multiple fibre populations within a voxel using the CSD framework. In the present work, FBA revealed fibre‐specific reductions in WM organisation in chronic mTBI patients compared to healthy controls, with significant decreases in FD and FDC observed in the left SLF‐II.

A strength of this work is the use of an acquisition protocol specifically optimized for FBA. Through the use of a multi‐shell acquisition with high *b* values (*b* = 5000 s/mm^2^) and high angular resolution (95 gradient directions), this protocol enhances sensitivity to the intra‐axonal compartment through attenuation of extra‐axonal water signals (Dhollander, Clemente, et al. 2021). At these high b‐values, the FD metric is approximately proportional to the *volume of the intracellular component of axons oriented in a given direction* (Dhollander, Clemente, et al. 2021; Genc et al. 2020; Raffelt, Tournier, Rose, et al. 2012). Decreases in FD are typically indicative of one of two underlying processes: a reduction in the volume of the intracellular component, which may reflect axonal degeneration or loss, or a decrease in the intracellular volume occupied by the same number of axons, suggestive of axonal atrophy (Raffelt, Tournier, Rose, et al. 2012; Raffelt, Dhollander, et al. 2017). Thus, the reductions in FD and FDC observed in our study likely reflect structural WM changes, such as axonal loss or reduced axonal calibre, that persist into the chronic phase of mTBI.

Our tract‐of‐interest analysis focused on large‐scale association, commissural, and projection pathways commonly implicated in mTBI (Lindsey et al. 2023; Shenton et al. 2012). The observed abnormalities in the SLF are consistent with prior DTI findings reporting axonal injury in this region across TBI populations (Alhilali et al. 2015; Geary et al. 2010; Kraus et al. 2007; Messé et al. 2012; Veeramuthu et al. 2015). Long‐range association fibres such as the SLF are positioned to bear significant mechanical stress during head acceleration and deceleration, making them more prone to injury (Bigler 2013; Chatelin et al. 2011). In a recent systematic review, Lindsey et al. (2023) identified 16 studies that performed tract‐of‐interest‐based analyses of the SLF, 11 of which found significant DTI‐related alterations over the course of injury. Many of these earlier studies have reconstructed as a single undifferentiated structure, despite its subdivision into three anatomically and functionally distinct branches (Wang et al. 2016). This study provided a more detailed analysis by investigating all three branches of the SLF, each of which is involved in distinct cognitive functions and may show unique vulnerabilities following mTBI (Nakajima et al. 2020; Thiebaut De Schotten et al. 2011).

The observed laterality effect in the left SLF‐II warrants consideration of potential hemispheric vulnerabilities in mTBI. Several prior DTI studies have similarly reported left‐lateralized WM abnormalities in association pathways in mTBI populations, including the SLF (e.g., Cubon et al. 2011; Mayer et al. 2010; Mito et al. 2022). The SLF has also been shown to exhibit leftward structural asymmetry in healthy individuals (Thiebaut De Schotten et al. 2011), and it has been hypothesized that GM regions/WM tracts with inherent asymmetry may be differentially susceptible to biomechanical injury (Dennis et al. 2022; Mito et al. 2022). In the present sample, there were no group differences in handedness and laterality indices of the SLF‐II. Therefore, the left‐sided FD/FDC reduction is unlikely to reflect group differences in laterality or a systematic shift in hemispheric asymmetry. Alternative explanations may relate to heterogeneity in injury mechanisms (Table 1), or biomechanics across patients (e.g., impact direction, translational/rotational forces and mechanical strain, head orientation at the time of injury), details of which were not available in our sample. Recent evidence has also suggested selective vulnerability of left frontal WM to secondary post‐concussive neuroinflammation and oedema (Pedersen et al. 2020), though further research is needed to determine whether these processes differentially affect left hemisphere structures. Ultimately, the mechanistic basis for left SLF‐II vulnerability in mTBI remains speculative and represents an important direction for future research.

### 6.3 Associations Between IIV and White Matter Organisation

The association between FBA metrics and IIV yielded an unexpected result. At the group level, patients demonstrated reduced FD and FDC, yet within the mTBI group greater IIV was linked to higher FDC in the left SLF‐II. At first glance, this finding appears contradictory, as we expected that reduced WM organisation would correlate with greater instability (MacDonald et al. 2009). In the following section, we will outline two possible explanations for this unexpected finding.

One possibility is that the finding reflects pathophysiological changes that lead to apparent increases in FD/FDC without necessarily improving functional efficiency. Prior work has shown that both increases and decreases in diffusion metrics may indicate deleterious changes (Lindsey et al. 2023). Pathophysiological processes, such as chronic neuroinflammation (Drieu et al. 2022; Simon et al. 2017), gliosis (Budde et al. 2011; Mannix et al. 2014; Stichel and Müller 1998), or changes in axon number and calibre (Donovan et al. 2014; Jafari et al. 1997) can alter the extracellular water balance within voxels, resulting in elevations in fixel‐based FDC. According to this explanation, patients with increased behavioural variability may also show more pronounced neuropathological changes within the SLF‐II. However, animal studies validating FBA metrics against gold‐standard histological assessments will be necessary to clarify the nature of these tissue changes (Wright et al. 2017).

An alternative explanation is that this unexpected finding reflects a compensatory reliance on frontoparietal networks at the functional level in mTBI patients. The SLF‐II is the major branch of the SLF connecting the dorsolateral prefrontal cortex with the parietal cortex and is a key component of the task‐positive frontoparietal network involved in attention and executive control (Nakajima et al. 2020; Thiebaut De Schotten et al. 2011). Task‐related fMRI studies have suggested that individuals with increased cognitive performance variability recruit frontoparietal control systems (including middle frontal and inferior parietal regions connected by the SLF‐II; Nakajima et al. 2020) to a greater extent during the performance of a Go/No‐Go task, reflecting the need for increased top‐down control (Bellgrove et al. 2004). In this context, the higher FDC observed in patients with elevated IIV could represent the structural support necessary for this compensatory recruitment of executive control circuits in those with more unstable performance, rather than more efficient WM organization. However, future multimodal work combining diffusion MRI with task‐based fMRI will be required to directly test whether structural variation in the SLF‐II corresponds to differential recruitment of frontoparietal control networks and concurrent performance fluctuations in mTBI patients.

Taken together, our findings suggest that structural variation in the SLF‐II may play a role in increased IIV in chronic mTBI. An important future direction will be to examine whether IIV can serve as a prognostic marker of WM health and long‐term behavioural outcome. Longitudinal research in cognitive aging has shown that increased IIV is predictive of future cognitive decline and conversion to mild cognitive impairment (Bielak et al. 2010; MacDonald et al. 2009). Although relatively few longitudinal studies have been conducted in mTBI, existing findings suggest that IIV is sensitive to post‐injury recovery processes and long‐term outcomes. For example, Vasquez et al. (2018) showed that IIV improved during the first year post‐injury but then declined again between 1 and 2 years post‐injury. We suggest that future studies should adopt longitudinal designs focused on tracking IIV over extended periods to clarify its prognostic value for recovery. EMA‐based *measurement burst designs* are especially well‐suited to address this question, as they can capture micro‐time variability (hours, days, weeks) across repeated macro‐time intervals (e.g., every 6–12 months over several years) (Sliwinski 2008; Stawski et al. 2019). Repeated neuroimaging taken at measurement burst intervals could in turn allow joint modelling of IIV and WM to test whether changes in IIV track with longitudinal changes in WM organisation.

## 7 Limitations and Future Directions

The present study has several limitations that warrant consideration. Most notably, the mTBI sample was small (*n* = 11), which limits the statistical power and generalizability of the findings to the broader mTBI population. Important to note, our EMA study comprised an intensive longitudinal design whereby each participant contributed a large number of observations through up to 30 days of EMAs and across repeated trials of a RT task. This design enabled us to derive robust IIV estimates despite the small sample size. In addition, the mTBI group was also heterogeneous in key clinical characteristics (as can be seen in Table 1), including prior mTBI history, missing GCS data (in two cases), and variables, such as time since injury and injury mechanism. While this diversity reflects the diversity of TBI presentations (Covington and Duff 2021), it may have introduced variability that influenced our between‐group comparisons and brain–behaviour associations. The small sample size also influenced key methodological choices in our analyses. For example, to mitigate type I error, we restricted our MRI analyses to three *a‐priori* tracts of interest rather than conducting a whole‐brain analysis, and we did not apply a correction for multiple comparisons on the behavioural variables given the exploratory nature of this work. Future research with larger samples and the use of whole‐brain analyses and more stringent statistical corrections will be required to reproduce our preliminary findings.

It is important to acknowledge that compliance varied among participants, with some completing as few as ~50% of the prompts. Notably, our study collected EMA data over a longer timeframe than many previous investigations of mTBI patients, which typically last 1 to 2 weeks (e.g., Pacella et al. 2018; Rabinowitz and Fisher 2020). Lower compliance rates are common in longer‐duration EMA studies such as ours, especially in those studies involving multiple daily prompts. A recent review highlighted several strategies to enhance engagement with EMA technology, including ongoing compliance monitoring, scheduling periodic check‐ins, and providing compliance‐based incentives (Heron et al. 2017). Nonetheless, our EMA protocol still achieved an acceptable compliance rate of 76%, which is consistent with prior EMA research on TBI populations with similar study durations (Burnett, Cobden, Firman‐Sadler, et al. 2025). For instance, Juengst et al. (2019) reported a compliance rate of 73.4% in a study involving once‐daily assessments over 8 weeks in mild‐to‐severe TBI patients.

Finally, the iSD provided a straightforward measure to capture IIV in our study. However, this approach has some inherent limitations. The iSD is a summary metric that reduces rich temporal data into a single variability score per person, which potentially masks important time‐varying patterns and contextual influences on IIV (Ram and Gerstorf 2009). Additionally, the iSD assumes that variability is stable across the measurement period and cannot account for systematic changes in both mean levels and variability over time (Mon et al. 2024; Ong et al. 2016). Future research could employ mixed effects location scale models (MELSMs), which were specifically developed for the analysis of EMA data (Hedeker et al. 2008, 2012). MELSMs can simultaneously model both mean performance levels and IIV as outcomes while accounting for time‐varying covariates, and would allow researchers to examine the influence of between‐person factors and within‐person processes on cognitive performance and variability.

## 8 Conclusion

This study is the first to investigate the relationships between cognitive IIV, symptomatology, and WM organisation in the chronic phase of mTBI. Using two complementary approaches, we identified both immediate and sustained disruptions to cognitive performance stability at different time scales, which were closely tied to changes in symptom severity and WM organisation. Overall, our findings suggest that cognitive variability is a persistent feature of mTBI and may reflect alterations in a major frontoparietal pathway implicated in attention and executive control. Our work highlights the potential value of utilising EMA and IIV metrics for exploring relationships between cognitive deficits and WM integrity in mTBI patients.

## Author Contributions

**J.B.:** conceptualization, formal analysis, methodology, investigation, visualization, writing – original draft, writing – review and editing; **A.C.:** methodology, writing – review and editing; **A.B.:** methodology, software development; **H.A.:** supervision; **J.F.D.D.:** methodology, supervision, writing – review and editing; **K.C.:** conceptualization, funding acquisition, methodology, project administration, resources, supervision, writing – review and editing.

## Ethics Statement

Ethical approval was obtained by the St Vincent's Hospital Human Research Ethics Committee (Ref: 034/22–72852) and Deakin University Human Research Ethics Committee (Ref: 2021‐129).

## Conflicts of Interest

The authors declare no conflicts of interest.

## Supporting information


**Table S1:** Correlations between EMA cognitive tasks, EMA RPQ‐13 symptoms, and RPQ‐16 total scores in the mTBI group.

## Data Availability

The data that support the findings of this study are available from the corresponding author upon reasonable request.
