# Supplementary material for: Association Between Intraindividual Variability in Cognitive Performance and White Matter Organisation in Chronic Mild Traumatic Brain Injury
Source: Hum Brain Mapp. 2025 Nov 1;46(16):e70394. doi: 10.1002/hbm.70394 (PMC12579384; doi:10.1002/hbm.70394)
Supplement: Supplementary file 1 — Table S1: Correlations between EMA cognitive tasks, EMA RPQ‐13 symptoms, and RPQ‐16 total scores in the mTBI group. [file HBM-46-e70394-s001.docx]

**Supplementary Table S1.**

*Correlations between EMA cognitive tasks, EMA RPQ-13 symptoms, and RPQ-16 total scores in the mTBI group.*

| **Measure** | **1** | **2** | **3** | **4** | **5** | **6** | **7** | **8** | **9** | **10** | **11** | **12** | **13** | **14** | **15** |
| --- | --- | --- | --- | --- | --- | --- | --- | --- | --- | --- | --- | --- | --- | --- | --- |
| 1. EMA RPQ-Cognition (iMean) | 1 |  |  |  |  |  |  |  |  |  |  |  |  |  |  |
| 2. EMA RPQ-Cognition (iSD) | .45 | 1 |  |  |  |  |  |  |  |  |  |  |  |  |  |
| 3. EMA RPQ-Emotion (iMean) | **.94***** | .32 | 1 |  |  |  |  |  |  |  |  |  |  |  |  |
| 4. EMA RPQ-Emotion (iSD) | **.85***** | **.70*** | **.80**** | 1 |  |  |  |  |  |  |  |  |  |  |  |
| 5. EMA RPQ-Somatic (iMean) | **.85***** | .32 | **.92***** | **.70***** | 1 |  |  |  |  |  |  |  |  |  |  |
| 6. EMA RPQ-Somatic (iSD) | .38 | **.86***** | .34 | .60 | .34 | 1 |  |  |  |  |  |  |  |  |  |
| 7. EMA RPQ-Total (iMean) | **.88***** | .21 | **.93***** | **.64*** | .**96***** | .21 | 1 |  |  |  |  |  |  |  |  |
| 8. EMA RPQ-Total (iSD) | **.70*** | .43 | .59 | .55 | **.66*** | .42 | **.70*** | 1 |  |  |  |  |  |  |  |
| 9. EMA 2-Back Task (iMean) | -.24 | -.01 | -.15 | .04 | -.20 | .24 | -.34 | -.31 | 1 |  |  |  |  |  |  |
| 10. EMA 2-Back Task (iSD) | .20 | .57 | .03 | .20 | .16 | .27 | .14 | .46 | **-.69*** | 1 |  |  |  |  |  |
| 11. EMA Card Matching (iMean) | -.18 | -.27 | .00 | .05 | -.10 | -.31 | -.13 | -.48 | .22 | -.39 | 1 |  |  |  |  |
| 12. EMA Card Matching (iSD) | -.10 | .39 | .00 | .04 | .17 | **.69*** | .02 | .18 | .15 | .18 | -.16 | 1 |  |  |  |
| 13. EMA Symmetry Span (iMean) | .02 | -.35 | .02 | -.28 | -.09 | -.07 | .02 | -.07 | .13 | -.50 | -.48 | .15 | 1 |  |  |
| 14. EMA Symmetry Span (iSD) | -.04 | .30 | .02 | .22 | .21 | .19 | .06 | .20 | .00 | .41 | .35 | .18 | -.90 | 1 |  |
| 15. RPQ-16 (Total Scores) | .76 | .15 | .82 | .56 | .92 | .16 | .91 | .63 | -.39 | .21 | -.05 | .16 | -.11 | .20 | 1 |

***Note***. * *p* < .05, ** *p* < .01, *** *p* < .001
